# The World Spider Trait database: a centralized global open repository for curated data on spider traits

**DOI:** 10.1093/database/baab064

**Published:** 2021-10-20

**Authors:** Stano Pekár, Jonas O Wolff, Ľudmila Černecká, Klaus Birkhofer, Stefano Mammola, Elizabeth C Lowe, Caroline S Fukushima, Marie E Herberstein, Adam Kučera, Bruno A Buzatto, El Aziz Djoudi, Marc Domenech, Alison Vanesa Enciso, Yolanda M G Piñanez Espejo, Sara Febles, Luis F García, Thiago Gonçalves-Souza, Marco Isaia, Denis Lafage, Eva Líznarová, Nuria Macías-Hernández, Ivan Magalhães, Jagoba Malumbres-Olarte, Ondřej Michálek, Peter Michalik, Radek Michalko, Filippo Milano, Ana Munévar, Wolfgang Nentwig, Giuseppe Nicolosi, Christina J Painting, Julien Pétillon, Elena Piano, Kaïna Privet, Martín J Ramírez, Cândida Ramos, Milan Řezáč, Aurélien Ridel, Vlastimil Růžička, Irene Santos, Lenka Sentenská, Leilani Walker, Kaja Wierucka, Gustavo Andres Zurita, Pedro Cardoso

**Affiliations:** Department of Botany and Zoology, Faculty of Science, Masaryk University, Kotlářská 2, Brno 611 37, Czechia; Zoological Institute and Museum, University of Greifswald, Loitzer Str. 26, Greifswald 17489, Germany; Department of Biological Sciences, Macquarie University, 6 Wally’s Walk, Sydney, NSW 2109, Australia; Slovak Academy of Sciences, Institute of Forest Ecology, Ľ. Štúra 2, Zvolen 960 01, Slovak Republic; Department of Ecology, Brandenburg University of Technology Cottbus-Senftenberg, Konrad-Wachsmann-Allee 6, Cottbus 03046, Germany; Laboratory for Integrative Biodiversity Research, Finnish Museum of Natural History LUOMUS, University of Helsinki, Pohjoinen Rautatiekatu 13, Helsinki 00014, Finland; Molecular Ecology Group (MEG), Water Research Institute (IRSA), National Research Council (CNR), Corso Tonolli, 50, Pallanza 28922, Italy; Department of Biological Sciences, Macquarie University, 6 Wally’s Walk, Sydney, NSW 2109, Australia; Laboratory for Integrative Biodiversity Research, Finnish Museum of Natural History LUOMUS, University of Helsinki, Pohjoinen Rautatiekatu 13, Helsinki 00014, Finland; Department of Biological Sciences, Macquarie University, 6 Wally’s Walk, Sydney, NSW 2109, Australia; Department of Botany and Zoology, Faculty of Science, Masaryk University, Kotlářská 2, Brno 611 37, Czechia; Department of Biological Sciences, Macquarie University, 6 Wally’s Walk, Sydney, NSW 2109, Australia; School of Biological Sciences, University of Western Australia, 35 Stirling highway, Crawley, WA 6009, Australia; Department of Ecology, Brandenburg University of Technology Cottbus-Senftenberg, Konrad-Wachsmann-Allee 6, Cottbus 03046, Germany; Department of Evolutionary Biology, Ecology and Environmental Sciences & Biodiversity Research Institute (IRBio), Universitat de Barcelona, Av. Diagonal 643, Barcelona 08028, Spain; Fundación Protectora Ambiental Planadas Tolima (FUPAPT, Tolima, Colombia; Instituto de Biología Subtropical (UNAM-CONICET), Puerto Iguazú, Argentina; Grupo de Investigaciones Entomológicas de Tenerife (GIET), C/ San Eulogio 15, 1º, La Laguna, Canary Islands 38108, Spain; Centro Universitario Regional del Este, Universidad de la República, Ruta 8 Km 282, Treinta y Tres, Uruguay; Department of Biology, Ecological Synthesis and Biodiversity Conservation Lab, Federal Rural University of Pernambuco, Dom Manuel de Medeiros, s/n, Dois Irmãos—CEP, Recife, PE 50710-270, Brazil; Department of Life Sciences and Systems Biology, University of Turin, Via Accademia Albertina, 13, Turin 10123, Italy; UMR CNRS 6553 ECOBIO, Université de Rennes 1, 263 Avenue du General Leclerc, Rennes 35042, France; Department of Botany and Zoology, Faculty of Science, Masaryk University, Kotlářská 2, Brno 611 37, Czechia; Laboratory for Integrative Biodiversity Research, Finnish Museum of Natural History LUOMUS, University of Helsinki, Pohjoinen Rautatiekatu 13, Helsinki 00014, Finland; Departamento de Biología Animal, Edafología y Geología, Universidad de La Laguna, La Laguna, Tenerife 38206, Spain; Division of Arachnology, Museo Argentino de Ciencias Naturales ‘Bernardino Rivadavia’—CONICET, Av. Ángel Gallardo 470, Buenos Aires C1405DJR, Argentina; Laboratory for Integrative Biodiversity Research, Finnish Museum of Natural History LUOMUS, University of Helsinki, Pohjoinen Rautatiekatu 13, Helsinki 00014, Finland; CE3C—Centre for Ecology, Evolution and Environmental Changes, Azorean Biodiversity Group and Universidade dos Açores, Angra do Heroísmo, Azores, Portugal; Department of Botany and Zoology, Faculty of Science, Masaryk University, Kotlářská 2, Brno 611 37, Czechia; Zoological Institute and Museum, University of Greifswald, Loitzer Str. 26, Greifswald 17489, Germany; Department of Forest Ecology, Faculty of Forestry and Wood Technology, Mendel University in Brno, Zemědělská 3, Brno 613 00, Czech Republic; Department of Life Sciences and Systems Biology, University of Turin, Via Accademia Albertina, 13, Turin 10123, Italy; Instituto de Biología Subtropical (UNAM-CONICET), Puerto Iguazú, Argentina; Institute of Ecology and Evolution, University of Bern, Baltzerstrasse 6, Bern 3012, Switzerland; Department of Life Sciences and Systems Biology, University of Turin, Via Accademia Albertina, 13, Turin 10123, Italy; Te Aka Mātuatua School of Science, University of Waikato, Private Bag 3105, Hamilton 3240, New Zealand; UMR CNRS 6553 ECOBIO, Université de Rennes 1, 263 Avenue du General Leclerc, Rennes 35042, France; Department of Life Sciences and Systems Biology, University of Turin, Via Accademia Albertina, 13, Turin 10123, Italy; UMR CNRS 6553 ECOBIO, Université de Rennes 1, 263 Avenue du General Leclerc, Rennes 35042, France; Division of Arachnology, Museo Argentino de Ciencias Naturales ‘Bernardino Rivadavia’—CONICET, Av. Ángel Gallardo 470, Buenos Aires C1405DJR, Argentina; Laboratory for Integrative Biodiversity Research, Finnish Museum of Natural History LUOMUS, University of Helsinki, Pohjoinen Rautatiekatu 13, Helsinki 00014, Finland; Crop Research Institute, Drnovská 507, Prague 6 CZ-16106, Czechia; UMR CNRS 6553 ECOBIO, Université de Rennes 1, 263 Avenue du General Leclerc, Rennes 35042, France; Biology Centre, Czech Academy of Sciences, Institute of Entomology, Branišovská 31, České Budějovice 370 05, Czechia; Grupo de Investigaciones Entomológicas de Tenerife (GIET), C/ San Eulogio 15, 1º, La Laguna, Canary Islands 38108, Spain; Island Ecology and Evolution Research Group, Instituto de Productos Naturales y Agrobiología (IPNA-CSIC), La Laguna, Tenerife, Canary Islands 38206, Spain; Department of Botany and Zoology, Faculty of Science, Masaryk University, Kotlářská 2, Brno 611 37, Czechia; Natural Sciences, Auckland War Memorial Museum, Parnell, Auckland 1010, New Zealand; Department of Biological Sciences, Macquarie University, 6 Wally’s Walk, Sydney, NSW 2109, Australia; Department of Anthropology, University of Zürich, Winterthurerstrasse 190, Zürich 8057, Switzerland; Instituto de Biología Subtropical (UNAM-CONICET), Puerto Iguazú, Argentina; Laboratory for Integrative Biodiversity Research, Finnish Museum of Natural History LUOMUS, University of Helsinki, Pohjoinen Rautatiekatu 13, Helsinki 00014, Finland

## Abstract

Spiders are a highly diversified group of arthropods and play an important role in terrestrial ecosystems as ubiquitous predators, which makes them a suitable group to test a variety of eco-evolutionary hypotheses. For this purpose, knowledge of a diverse range of species traits is required. Until now, data on spider traits have been scattered across thousands of publications produced for over two centuries and written in diverse languages. To facilitate access to such data, we developed an online database for archiving and accessing spider traits at a global scale. The database has been designed to accommodate a great variety of traits (e.g. ecological, behavioural and morphological) measured at individual, species or higher taxonomic levels. Records are accompanied by extensive metadata (e.g. location and method). The database is curated by an expert team, regularly updated and open to any user. A future goal of the growing database is to include all published and unpublished data on spider traits provided by experts worldwide and to facilitate broad cross-taxon assays in functional ecology and comparative biology.

Database URL: https://spidertraits.sci.muni.cz/

## Introduction

With almost 50 000 species described to date (1), spiders are among the most diverse orders of terrestrial arthropods (2). Spiders rank among the most dominant arthropod predators in a huge variety of ecosystems and therefore provide important ecosystem services, such as biological control (3, 4) and bio-indication (5). They are also potentially an important source of molecules to be used in new biotechnologies and human medicine (6, 7). In addition to these uses, spiders provide suitable models to test the breadth of ecological and evolutionary hypotheses (8–10).

Successful use of spiders for research and environmental assessments is based on knowledge of traits (morphological, ecological, physiological or behavioural characteristics), which characterize responses to environmental conditions and both change and define the effects of spiders on ecosystem functioning (10). Assembling trait values for species in a community is laborious because, for some traits and species, this information either does not exist or is not easily available as it is hidden in old publications (often not in English), unpublished records, technical reports or even field notes. Although difficult to access, the data available are extensive as research on spiders has covered a huge diversity of topics for over 200 years (11). Data on spider traits continues to be generated on a daily basis, most of it being used in individual publications or retained in unpublished datasets. Trait data are stored in different places and forms, and most data that originated before the use of personal computers are only available from printed publications. More recently, collected data have often been stored in digital form in different repositories (from personal computers to data archive servers), but it is often difficult to compile and standardize datasets with different formats and completeness of metadata, which are necessary for leveraging data for common purposes as pointed out in the concept of Essential Biodiversity Variables (12, 13).

Trait databases already exist for a number of taxonomic groups, such as plants (14), corals (15), reptiles (16), copepods (17) and ground beetles (18), with a similar aim to accumulate and organize available data in a single repository. The success of such databases can be seen in their frequent use by many scholars (19). A general database of spider traits has not yet been developed. However, a range of spider traits can currently be found in several online resources, for example, the body size of European species (20), cytogenetic data (21), protein toxins of spiders (22), habitat and phenology of British (http://srs.britishspiders.org.uk/) and Czech spiders (http://arachnobaze.cz/) and various traits of ground-dwelling spiders (https://portail.betsi.cnrs.fr).

A single database that accommodates all trait data would enable scientists to investigate spiders more effectively and to perform large-scale comparative analyses (23–29). A trait-based approach has the advantage that some investigations (e.g. bio-indication) can be performed even when the taxonomic identity is missing or inaccurate (using morphospecies, for example) (30). Using traits, instead of taxonomic information, also allows for a comparison of community patterns and responses across regions with different species pools (31). For these purposes, it is important that trait data are available in appropriate quality and quantity and have broad taxon and regional coverage. Overcoming these barriers will foster collaboration among arachnologists and other researchers that aim for multi-taxa analyses (24, 32, 33).

Recently, Lowe *et al.* (10) initiated the establishment of a centralized database that aims to cover all spider traits and store data in a single place under FAIR (findable, accessible, interoperable and reusable) principles (34). Lowe *et al.* (10) built the foundation of such a database by detailed coverage of the trait definition, their standardization, input data types, database governance, geographical coverage, accessibility, quality control and sustainability. Furthermore, Lowe *et al.* (10) recognized that the unification of the trait records can only be accomplished by careful examination of the data during the validation procedure.

**Figure 1.**
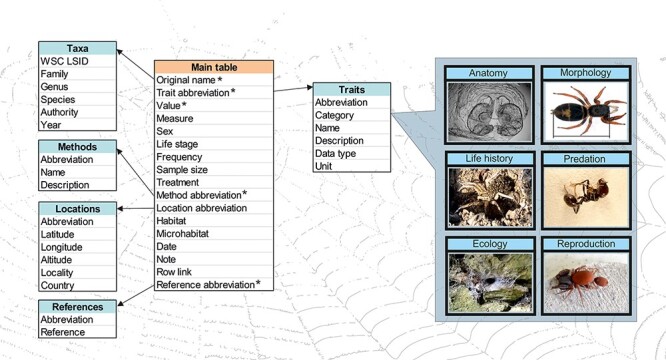
A scheme of the database structure. There is the main table connected to five metadata tables. * marks mandatory variables. Examples of trait categories are given on the right. Photos: S. Pekár.

Following the initiative (10), here, we present a curated global database that follows the FAIR principles and hosts a variety of traits recorded for spiders (Figure 1). With the potential to grow indefinitely, we have already collected data for more than 7000 spider taxa so far. The database has two main goals: (i) to collect and curate trait data on spiders from different sources, either (un)published or to be published in the future, and (ii) to provide public access to these data under a CC BY licence, facilitating their widespread use by researchers.

## Methods

### Definitions

We adopted a broad definition of traits for inclusion in our database: any measurable phenotypic (i.e. morphological, ecological, physiological and behavioural) characteristic of an individual or taxon. This may also include ‘pure’ (heritable) traits (35), as well as the response to environmental conditions or a treatment (36, 37). Traits can be either quantitative (continuous, integers and proportions) or categorical (qualitative, binary and ordinal). Trait values can represent individual-level measurements (single observation) to higher taxonomic (species-, genus- and family-) level measurements (aggregates), often recorded as a statistic (mean, median, minimum and maximum). We do not consider descriptive molecular data (such as DNA or protein sequences) or faunistic records to be traits, unless these contain reference to some trait (e.g. habitat type), as these have already established repositories, such as GenBank® or the Global Biodiversity Information Facility.

The definition of specific traits (including units for numerical traits or eligible values for categorical traits) was adopted from widely used definitions in a variety of published papers on spiders. To achieve semantic interoperability, each trait is described by standardized terms (Table S1). Two types of ontologies, describing the process of data collection and the traits themselves, were implemented during the development of the database structure, as suggested by Kissling *et al.* (12). The process of measurement, that is, details of data collection, is provided as metadata, and the trait measured is given in the main table (see below).

To increase the interoperability of this database with other databases, the next step in the update of the database will be setting up an expert team to develop ontologies, detailed vocabularies and a hierarchical structure for all traits. Some traits thus might be redefined. This will not affect the current content but will prepare space for a harmonized collection of future data.

### Database structure

**Figure 2.**
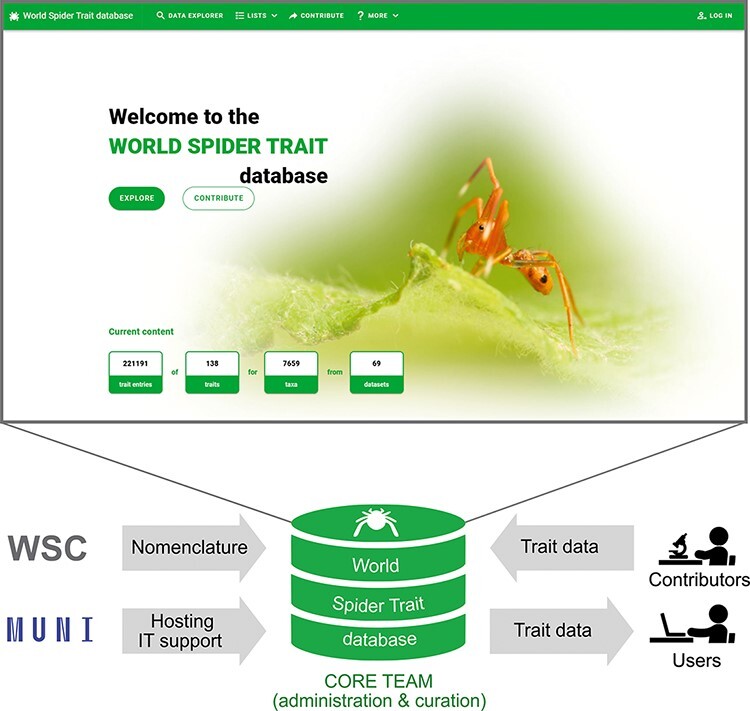
The scheme of the World Spider Trait database application, depicting the role of contributing bodies and the frontpage of the webpage (https://spidertraits.sci.muni.cz/, accessed on 5 March 2021). WSC stands for World Spider Catalog, MUNI stands for Masaryk University.

We developed an online application and architecture called the World Spider Trait database, currently in version 1.0 (https://spidertraits.sci.muni.cz/), to store and retrieve trait data on spider species (Figure 2). The database is able to accommodate traits measured at any taxonomic level. As many trait values show variation (phenotypic plasticity) as a response to varying conditions, each trait record can be accompanied by extensive metadata, describing the conditions under which it was measured (such as treatment, sampling method, geographic location, habitat and date). The database was built to meet the FAIR principles: it is available at a public domain under an open-access licence in a machine-readable format. This is enhanced by comprehensive online search options and export capabilities.

The database has multi-layered structure. It is composed of a main table (Figure 1), including five mandatory variables, namely (i) Original species name (taxon name as reported in the original source), (ii) Trait abbreviation (unique abbreviation of each trait), (iii) Trait value (measured value of a trait), (iv) Method abbreviation (unique abbreviation of each method used to measure a trait) and (v) Reference abbreviation (unique abbreviation of each source). Several other variables are optional, namely WSC LSID (unique taxon identifier taken from the World Spider Catalog), Trait category (see below), Trait name, Trait description, Trait data type, Trait unit, Measure (type of the measured value), Life stage (ontogenetic stage), Sex, Frequency (relative frequency of occurrence), Sample size (total number of observations per record), Treatment (treatment conditions), Method name (see below), Method description, Location abbreviation (unique identifier of a location), Latitude, Longitude, Altitude, Locality (the name or description of the place), Country, Habitat (habitat type according to a local classification), Microhabitat, Date, Note (any note related to a record), Row link (unique identifier of related measurements) and Reference (full reference). For a detailed description of each variable and examples, see Table 1.

**Table 1.** Content of the template file. For each variable, there is its name, description and eligible values

Variable name	Description	Eligible values or examples
WSC LSID	Taxonomic identifier (URN) from the World Spider Catalog	(urn:lsid:nmbe.ch:spidersp:033381)
Original name*	Taxon name as reported in the original source	(Linyphiidae, *Zodarion* sp., *Pimoa rupicola*)
Trait abbreviation*	Abbreviation (see Table S1)	(indu)
Value*	Measured value of a trait	(110)
Measure	Type of the measured value	Single observation, mean, median, min, max, description
Sex	Sex	Female, male, both, unknown
Life stage	Ontogenetic stage	Egg, larva, juvenile, adult, all
Frequency	Relative frequency of occurrence	(0.43)
Sample size	Total number of observations per record	(45)
Treatment	Treatment and conditions at which it was measured	(Effect of a pesticide, type of prey, wavelength, temperature)
Method abbreviation*	Abbreviation (see Table S2)	(ptf)
Latitude	The geographic latitude (in decimal degrees or other widely used formats)	(45.74, −37.22285)
Longitude	The geographic longitude (in decimal degrees or other widely used formats)	(102.478922, −0.4767)
Altitude	Altitude of the location (above sea level in meters)	(567)
Locality	The name or description of the place	(Municipality of Helsinki, small hill close to the river, Mount Fuji)
Country	The standard code for the country	According to ISO 3166 (CZ, IT, BR, CZE)
Habitat	Habitat type according to a local classification, such as European Nature Information System (EUNIS)	(Pine forest, grassland, cave)
Microhabitat	Microhabitat type	(Under stones, ground, canopy)
Date	The date-time or interval	(March 8, 1963T14:07-February 20, 0600, 2009T08:40Z, August 29, 2018T15:19-3:19pm, 1906-06, 1971)
Note	Any note related to information provided	(Habitat classification, experimental procedure)
Row link	Unique identifier marking-related data (same individuals)	(a1)
Reference*	Full reference of the published or unpublished data	( Journal: Elias DO, Hebets EA, Hoy RR & Mason AC. 2005. Seismic signals are crucial for male mating success in a visual specialist jumping spider (Araneae: Salticidae). Animal Behaviour 69 (4): 931–938. Book: Preston-Mafham R. 1990. The Book of Spiders and Scorpions. London, Quantum Books. Book Chapter: Nentwig W. 1987. The prey of spiders. In Nentwig W (Ed.), Ecophysiology of Spiders. Berlin, Springer-Verlag, pp. 249–263. Website: Nentwig W, Blick T, Bosmans R, Gloor D, Hänggi A, Kropf C (2021) Spiders of Europe. Online at https://www.araneae.nmbe.ch. Unpublished: Michalko R, pers. comm.

Mandatory variables are indicated by an asterisk (*).

Eligible values are predefined only for some variables. Examples are given in parenthesis.

In the backend of the application, there are five additional metadata tables (extensions) that provide auxiliary information: (i) Taxa, (ii) Locations, (iii) Traits, (iv) Methods and (v) References. The Taxa table includes valid species or subspecies name, genus, family, LSID (taxonomic identifier automatically retrieved from the World Spider Catalog (1), taxonomic authority and year. The content of this table is automatically updated on a weekly basis from the spider nomenclature information available in the World Spider Catalog (1), which contains valid Latin names and synonyms. Morpho-species do not have valid species names, thus higher level categories (e.g. genus) are used, optionally accompanied by additional information provided by the uploader in the Note field. The Locations table includes country code, country name, locality name, coordinates and its abbreviation. The Traits table contains trait name, category, description, data type, unit and its abbreviation. The Methods table includes method name, description and its abbreviation. References table includes full reference and its abbreviation. For more details see Table 1.

We defined 175 traits that are currently grouped into 12 categories according to the discipline (Anatomy; Biomechanics; Communication; Cytology; Defence; Ecology; Life-History; Morphology; Morphometry; Physiology; Predation and Reproduction) (Table S1). Information on the way a trait was measured is described in the Methods table. The provision of this metadata is mandatory during upload to ensure comparability of data. The Methods list includes field collection techniques, as well as laboratory methodologies. Currently, there are 37 methods defined (Table S2). The included pre-defined traits, categories and methods are meant to cover the majority of traits and methodologies in spider research. However, the architecture of the database is flexible enough that further traits, categories and methods can be added in the future to accommodate new trait types and novel methodologies.

This database is hosted, developed and maintained at the Department of Botany and Zoology of Masaryk University in collaboration with the University IT centre. It is connected to the World Spider Catalog (1), and administered and curated by the core team members (Figure 2).

### Data upload procedure

Upon collection, the data must be harmonized. Before a dataset can be submitted to the database, the data must be in a valid format (for a detailed description, see https://github.com/oookoook/spider-trait-database/blob/master/docs/template.md). For this purpose, we developed an MS Excel spreadsheet (Template) that should fit the great majority of trait types with predefined columns. The spreadsheet was designed to enable easy data manipulation by classical statistical software, such as R (38). The template can be downloaded from the World Spider Trait database webpage (https://spidertraits.sci.muni.cz/contribute). It contains 31 columns, some of which are mandatory, so they must be filled with appropriate numerical or character values. Eligible values for all columns can be found in the header of each variable in the List of Traits (Table S1) and List of Methods (Table S2). If the input trait or method is not already defined, the contributor should provide all of the following information to create a new trait or method: trait category, trait name, trait description, trait data type and trait unit in the case of missing traits or method name and method description in the case of missing methods. Similarly, for references, the contributor either provides an abbreviation of a reference if it is in the List of References or a full reference. Unpublished data are referenced as personal observations.

The data in the template then needs to be saved either as an .xls(x) or a comma-delimited .csv file, and the file should be encoded as UTF-8 to assure compatibility with special (regional) characters. Once the template is uploaded, the contributor must approve it using the tools within the web application.

### Software used

The code of the web application is stored at GitHub (https://github.com/oookoook/spider-trait-database) and is available under the GNU GPL v 3.0. The phylogenetic tree was produced using functions within ape package (39) within R (38).

## Results and discussion

### Data records

Integration of data from different sources was based on standardization and harmonization. This involved the conversion of trait values to comparable units/trait, use of controlled vocabulary in the definition of traits, standardization of eligible character values and use of single spreadsheet format. Each record was accompanied by licence information and the original source.

Currently, both published (from more than 1000 publications) and unpublished data from diverse study designs (both descriptive and experimental) are included in the database, with the citation of the original source. So far, 70 datasets have been contributed, with a total number of more than 221 000 records belonging to more than 7500 taxa. Of these, 40 datasets (34.1% of records) are unlocked (i.e. freely accessible without user registration). The remainder (i.e. embargoed datasets) are previously unpublished data compilations and can be viewed and downloaded by registered users only to ensure applicable authorship credits (see ‘Usage Notes’). Registration and data usage are free under a CC BY licence. Embargoed data compilations may eventually become unlocked (e.g. once these have been used in published studies).

**Figure 3.**
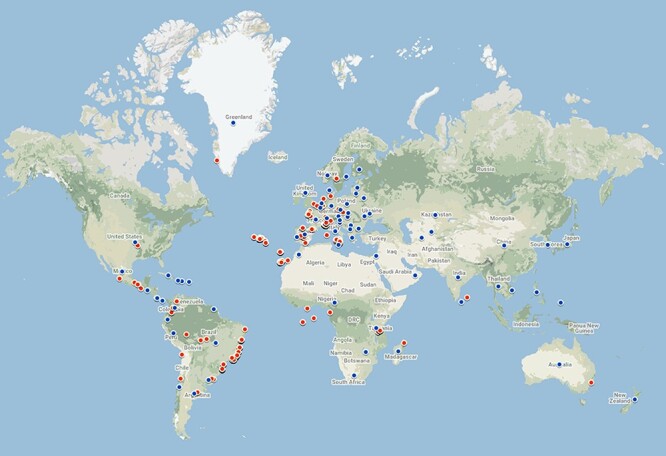
Geographic coverage of the data currently in the database. Red points represent geo-referenced records, while blue points are country centroids (for records that do not have an exact geographical reference). There are records from 70 countries and 479 locations. The map was created using Google Maps.

Geographical coverage of the database is global, but, currently, there are more records from Europe and South America than from other continents (Figure 3)—a typical bias in biodiversity research (40). Data on taxa from North America, Africa and Asia are represented by very few records. The great majority of records available now come from Europe. Specifically, 20 datasets (66.1% of records) concern European species. These include data on body size (2024 species), light and moisture preferences (1949 species), guild classification (1017 species) and conservation status (1557 species). In terms of traits, anatomical, behavioural and physiological data are largely missing.

**Figure 4.**
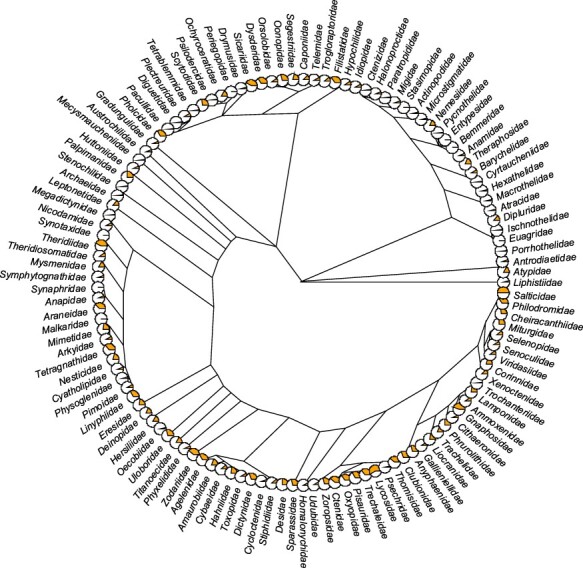
Trait coverage mapped on the tree. The tree is on the family level (composed of 121 families) with the proportion of the total number of traits (orange) displayed as pie charts (the fuller the pie, the more the traits). The tree was constructed based on the recent phylogeny of spiders (42). Five families (Hexurellidae, Mecicobothriidae, Megahexuridae, Microhexuridae and Myrmecicultoridae) were omitted because their position in the tree is not known.

**Figure 5.**
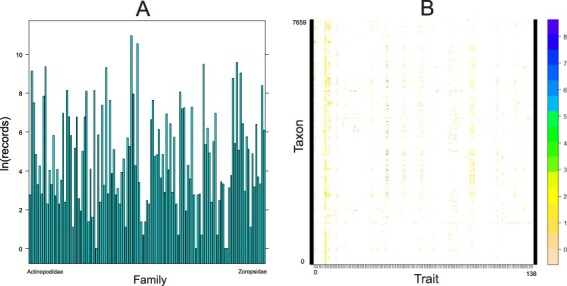
Quantitative content of the database. A. Number of records (logarithmically transformed) for each family included in the database, arranged alphabetically. B. The taxon by trait matrix representing the completeness. The most complete traits include body length (64% of taxa), followed by cephalothorax length (23%) and cephalothorax width (19%). Dots represent logarithmically transformed number of records per taxon. Taxon includes one of the following: subspecies, species, genus or family.

As for the taxonomic coverage, of 129 known spider families (1), only 2 (Euctenizidae and Penestomidae) have no records in the database so far (Figure 4). Several families (e.g. Gnaphosidae, Lycosidae, Salticidae, Sicariidae and Theridiidae) have data for more than 40% of the 138 traits, but 38 families still have fewer than 5% of all traits covered. As for the number of records per family, most records come from the most speciose families, namely Linyphiidae, followed by Lycosidae, Theridiidae and Salticidae (Figure 5A). Because not every trait has been measured for every taxon, the taxon × trait matrix is highly incomplete (2.82% completeness; Figure 5B). This is to be expected for a highly diverse and severely understudied taxonomic order. With respect to sex/stage, there are 33.6% records for adult males, 55.8% adult females and 8.6% for juveniles.

The content of the database reflects real historical differences among geographic areas and disciplines. The database thus can be used to identify gaps and help to prioritize future areas for investigation to achieve more complete sets of records. To fill these gaps, we plan to encourage contributions from specific areas, traits and trait categories in the future. This can include the collection of data from other repositories, extraction of data from publications and archiving currently produced data. We will also ask curators of specialized spider trait databases to provide their data to be centrally stored here. Since many funders and journals now require data to be made publicly available, the database can be used as a permanent data archive option (an alternative to, e.g. Dryad or Figshare), provided that each contributed dataset meets the standards of the database format, which allows efficient reuse and synthesis. Each dataset obtains a unique URL and, in near future, it will be associated with a DOI provided by DataCite. In the future, we expect to mainly gather data on new traits and new taxa and would like to encourage colleagues to contribute their datasets of both published and unpublished data. A coordinated effort is needed to achieve this goal.

To promote the process of data collection, we invite arachnologists to download the template and use it for data storage on their personal computers. At the same time, we ask arachnologists to get used to the vocabulary of the database, adopt the definition of the traits that are used here (or suggest alternatives) and develop protocols that follow the same standards. This will markedly enhance the integration of their datasets into the database.

### Data validation

Validation is performed at several steps during submission in order to retain only high-quality records.

First, a contributor is advised to search through the current database content in order to ensure that such (exact) data are not already included for the taxon/taxa under investigation. It is also useful at this point to check whether the proposed trait(s) and method(s) are already defined. Contributors become eligible to upload their dataset after requesting registration from the administrator.

To upload a new dataset, a contributor must specify the name of the dataset, their full name and email address. In addition, a contributor can specify the authors of the dataset and author emails and mark whether the data can be immediately accessed or are under an embargo and add any note. Then, the dataset sheet is created and the contributor is able to upload the data. The data is then imported to the temporary cache. During the upload process, the web application checks the presence of eligible values in the variables (Original name, Trait abbreviation, Value, Measure, Sex, Life stage, Frequency, Sample size, Method abbreviation, Latitude, Longitude, Altitude, Country, Date and Reference) and identifies duplicate records. Invalid records are highlighted to facilitate corrections. The taxonomy check includes existence of the name and match with a current valid name according to the World Spider Catalog (1).

At this stage, the contributor can view the dataset and must edit invalid cells in order to comply with the database requirements. Editing is done using the web application tools. When the contributor completes all changes and the dataset is valid, it can be sent to the administrator or editor for review. The contributor can include a message to the editor when submitting the dataset for review, in which the contributor can explain any problems they had encountered while editing the dataset.

The administrator or editor is informed of a new dataset submission by an email. The dataset enters a second validation phase, which can only be done by the administrator or editor. The administrator or editor must add new trait(s) and method(s) to the database, check for additional errors, such as extreme (unlikely) values of traits (e.g. resulting from typos and wrong digit separator), imprecise definition of new traits and methods or an incorrect format of references. Once the dataset is validated by the administrator or editor, it is published in the database. This means that all the data are transferred from the temporary import cache to the main database and become available to the general public, unless embargoed. If the administrator or editor observes any problems, the dataset is rejected and sent back to the contributor with an email containing a description of the problem(s) to be fixed. Any dataset can be *posthoc* corrected/altered by the administrator or editor without contributors’ consent.

### Data usage

A user can view the whole content of the database using the Data Explorer within the online application. In the Data Explorer, the user can apply filters (Family, Genus, Species, Original name, Trait category, Trait, Method, Location, Country, Dataset, References and Row links) to display selected content. The result can be displayed in a spreadsheet or in bar figure window. Selected data can then be downloaded in a .csv or .xlsx format. If the selected data contain data from datasets under embargo, the user is given a warning. In order to download embargoed data, the user has to send a request to the administrator or editor, who will then contact the dataset authors. Data with embargo can be download only after receiving login data.

In addition, the database provides an Application Programming Interface (API) to allow access to data via web platforms or software. An R package, named ARAKNO (41), with few easy-to-use functions that allow downloading and pre-processing data from the database, is now available. Resulting data frames can then be analysed with a variety of tools available in R (38). Access of the embargoed data via API requires login as well.

As the trait value data can be a mixture of various statistics, it is important that the user checks the ‘Measure’ variable of each record and adopts appropriate procedures prior to analysis. Furthermore, due to inherent variation in most trait values, the user must consider conditions (such as habitat, altitude and treatment) under which it was measured. Not all conditions (e.g. hunger state and mating status) are recorded in the auxiliary variables; thus, the user is strongly advised to study the original publication.

A number of traits included in this database are candidates of Essential Biodiversity Variables proposed by the Group on Earth Observations Biodiversity Observation Network (12, 13). The traits are recorded with many metadata and thus allow quantification of intra-specific variation with respect to environmental conditions, space and time. These traits can be of societal relevance, as they can be used in the study spread of invasive species or biodiversity change.

Although the use of data is free, users are strongly encouraged to contribute their data, particularly if they have not contributed yet, following the simple ‘first give, then take’ principle. Only by these means will the database grow in quantity and frequency of use.

Contained data are publicly available under a Creative Commons Attribution license (CC BY 4.0) so that anyone can use received data under the condition of appropriate citation of this publication. In the case of datasets that have not been published and are under embargo, the user must agree with the dataset contributor on the conditions of use. Typically, this should include citation (URL or DOI) of the specific dataset in addition to the database citation.

## Supplementary Material

baab064_Supp
