# Supplementary material for: The World Spider Trait database: a centralized global open repository for curated data on spider traits
Source: Database (Oxford). 2021 Oct 20;2021:baab064. doi: 10.1093/database/baab064 (PMC8517500; doi:10.1093/database/baab064)
Supplement: baab064_Supp [file baab064_supp.zip › tableS1.docx]

**Table S1.** List of traits, their description, types of data, units and eligible values, arranged alphabetically within trait categories.

| **Abbr.** | **Category / Name** | **Description** | **Data type** | **Unit** | **Eligible values** |
| --- | --- | --- | --- | --- | --- |
|  | Anatomy |  |  |  |  |
| brsi | Brain size | Volume of central nervous system | real number | mm^3^ |  |
| cuth | Cuticle thickness | Thickness of a cuticle on a body part | real number | mm |  |
| nugl | Number of silk glands | Number of any silk glands per spinneret | integer |  |  |
| prsy | Posterior respiratory system | Type of posterior respiratory system | character |  | Booklungs; tubular tracheae; lamella; absent |
| scle | Sclerotisation | Enhanced sclerotisation of prosoma | character |  |  |
| sigl | Silk gland size | Volume of any of the silk glands | real number | mm^3^ |  |
| stfo | Sperm transfer form | State in which sperm is transferred to the female | character |  |  |
| vgsi | Venom gland size | Volume of a venom gland | real number | mm^3^ |  |
|  | Biomechanics |  |  |  |  |
| adhe | Web adhesion | Adhesion of capture thread | real number | MPa |  |
| cspd | Climbing speed | Climbing speed (moving on a slope) | real number | cm/s |  |
| rspd | Running speed | Running speed (moving horizontally) | real number | cm/s |  |
| stra | Silk strain | Engineering strain of silk | real number | mm/mm |  |
| stre | Silk strength | Engineering strength of silk | real number | Mpa |  |
| toug | Silk toughness | Toughness of silk | real number | MPa |  |
|  | Communication |  |  |  |  |
| coop | Colouration of opisthosoma | Reflectance of dorsal side of opisthosoma at a certain wavelength (specify in Treatment variable) | real number | % |  |
| copr | Colouration of prosoma | Reflectance of dorsal side of prosoma at a certain wavelength (specified in Treatment variable) | real number | % |  |
| fred | Dominant frequency | The peak frequency of the sound produced | real number | Hz |  |
| freq | Lower frequency range | The minimum frequency of the sound produced | real number | Hz |  |
| freu | Upper frequency range | The maximum frequency of the sound produced | real number | Hz |  |
| soun | Sound production | Mechanism of sound production | character |  | Drumming; stridulation |
| sour | Sound source | Organs used to produce sound | character |  | Chelicera/pedipalp; chelicerae; prosoma/abdomen; leg/leg |
|  | Cytology |  |  |  |  |
| chrn | Chromosome number | Diploid number of chromosomes | integer |  |  |
| chrs | Sex chromosome system | The sex-chromosome system | character |  | X0; XX0 |
| chrt | Chromosome morphology | Type of chromosomes according to the position of the centromere | character |  | Acrocentric; holocentric; metacentric |
|  | Defence |  |  |  |  |
| modl | Model | Model imitated by species using camouflage and mimicry | character |  | Ant; beetle; wasp; snail; twig; branch; flower; etc. |
| prde | Primary defence | A strategy used prior to being detected by a predator | character |  | Cryptic (background matching); Cryptic (disruptive coloration); Cryptic (countershading); Aposematic; Batesian mimicry; Camouflage; Müllerian mimicry |
| pred | Predator | Taxonomical classification of a predator | character |  |  |
| retr | Retreat | Type of a retreat used to avoid predation | character |  | On web; in grass; under bark; silk sac; burrow; other |
| sede | Secondary defence | A strategy used after being detected by a predator | character |  | Death feigning; rapid escape; threatening posture; dazzle camouflage; startle; chemical deterrents; colour change; sound production |
|  | Ecology |  |  |  |  |
| balo | Ballooning | Developmental stage that disperses by ballooning | character |  | Juvenile; adult |
| circ | Circadian activity | Hours of a day when the species is active, i.e. foraging, mating, web-building | character |  | Diurnal; nocturnal; 1-24 |
| disp | Dispersal time | Months at which dispersal occurs | character |  | January; February; March; April; May; June; July; August; September; October; November; December |
| girl | Global IUCN Red List category | Category of risk according to the global IUCN Red List guidelines | character |  |  |
| habi | IUCN habitat | Habitat type according to the global IUCN classification | character |  | Forest; Savanna; Shrubland; Grassland; Wetlands; Rocky areas; Caves and Subterranean Habitats; Desert; Freshwater; Coastal; Urban, Agricultural; Other |
| halo | Habitat local CZ | Habitat type according to Czech habitat classification according to Chytrý M, Kučera T, Kočí M, Grulich V & Lustyk P. 2010. Habitat Catalogue of the Czech Republic. 2nd ed. Praha: Agentura ochrany přírody a krajiny ČR. | character |  |  |
| lepr | Legal protection | Legal protection in national or subnational legislations | character |  |  |
| ligh | Light | Light gradient according to Entling W, Schmidt MH, Bacher S, Brandl R & Nentwig W. 2007. Niche properties of Central European spiders: Shading, moisture and the evolution of the habitat niche. Global Ecology and Biogeography 16(4): 440–448. | real number |  |  |
| lig2 | Light 2 | Light gradient according to Buchar J & Růžička V. 2002. Catalogue of spiders of the Czech Republic. Praha, Peres. | character |  |  |
| mdl | Regional non-IUCN Red List category | Category of risk according to the regional guidelines (non-IUCN) | character |  |  |
| miha | Microhabitat | Habitat where species occurs | character |  | among stones; bare ground; herbs; bushes; foliage; litter |
| moi1 | Moisture 1 | Moisture gradient according to Entling W, Schmidt MH, Bacher S, Brandl R & Nentwig W. 2007. Niche properties of Central European spiders: Shading, moisture and the evolution of the habitat niche. Global Ecology and Biogeography 16(4): 440–448. | real number |  |  |
| moi2 | Moisture 2 | Moisture gradient according to Buchar J & Růžička V. 2002. Catalogue of spiders of the Czech Republic. Praha, Peres. Quantification of preference, where 1=preferred value, 0.5=primary value, 0.1=marginal value, can be given in Frequency variable. | character |  | Dry; semi-humid; humid |
| ovws | Overwintering stage | Developmental stage that overwinters | character |  | Egg; larva; juvenile; adult |
| phen | Phenology | Months at which adult stage occurs | character |  | January; February; March; April; May; June; July; August; September; October; November; December |
| rasi | Range size | Area of the species distribution range | real number | km^2^ |  |
| regl | Regional IUCN Red List category | Category of risk according to the regional IUCN guidelines | character |  |  |
| soci | Social degree | Degree of sociality | character |  | Solitary; subsocial; colonial; quasisocial; social |
| strt | Stratum | Horizontal stratum occupied | character |  | Underground; ground; herb layer; shrub layer; tree trunks; canopy; wall |
| suaf | Subterranean affinity | Degree of subterranean affinity | character |  | Troglobiont; troglophile |
| urha | Urban habitat | Affinity for urban habitats: % of urban habitats (i.e. impervious surfaces) in a buffer of 1600 m of radius around the sampling point | real number | % |  |
|  | Life History |  |  |  |  |
| indu | instar duration | Number of days spent in a certain ontogenetic stage (egg, larva, or instar) at a certain temperature (specified in Treatment variable) | integer | days |  |
| inst | Number of instars | Total number of instars, beginning with the first free instar and ending with the adult stage | integer |  |  |
| lonv | Longevity | Number of days from hatching to death | integer | days |  |
| mort | Mortality | Mortality either natural or due to any treatment | real number | % |  |
| sexr | Sex ratio | Number of males divided by the number of females | real number |  |  |
| surv | Survival | Proportion of surviving individuals | real number | % |  |
|  | Morphology |  |  |  |  |
| crib | Cribellum | Presence of functional cribellum and calamistrum (e.g., present) | character |  |  |
| ente | Entelegyne | Presence of epigyne in females (e.g., present) |  |  |  |
| eyes | Eye number | Total number of eyes | integer |  |  |
| flat | Body flattening | Significantly flattened body as an adaptation to shelter in crevices | character |  |  |
| nusp | Spine number | Number of erectable spines (macrosetae) on the prolateral side of leg I (that play a role in the formation of the capture basket) | integer |  |  |
| scoa | Scopula area | Area of scopula hairs on leg segments | real number | mm^2^ |  |
| scod | Scopula density | Number of scopula hairs per area on a leg segment | real number | mm^2^ |  |
| spin | Spinnerets | Total number of functional spinnerets | integer |  |  |
|  | Morphometry |  |  |  |  |
| abhe | Abdomen height | Opisthosoma height at highest point | real number | mm |  |
| able | Abdomen length | Opisthosoma length from anterior to posterior along longitudinal axis (excl. petiole and spinnerets) | real number | mm |  |
| abwi | Abdomen width | Opisthosoma width at widest point | real number | mm |  |
| aled | ALE | Diameter of one anterior lateral eye | real number | mm |  |
| alsl | Spinneret ALS | Total length of anterior lateral spinneret (from base to tip) | real number | mm |  |
| amed | AME | Diameter of one anterior median eye | real number | mm |  |
| bodm | Body mass | Body mass (in a normal nutritional condition) | real number | g |  |
| bole | Body length | Total body length (from carapace frontal, excl. chelicerae, to opisthosoma posterior, excl. spinnerets) | real number | mm |  |
| cehe | Cephalothorax height | Height of prosoma at the highest point (from sternum most ventral to carapace most dorsal) | real number | mm |  |
| cele | Cephalothorax length | Length of prosoma (carapace) along the longitudinal body axis | real number | mm |  |
| cewe | Cephalothorax width | Width of prosoma (carapace) at the widest point | real number | mm |  |
| chle | Chelicerae basal part (paturon) length | Length of cheliceral base segment (paturon) along external margin | real number | mm |  |
| cox1 | Coxa I length | Coxa length of leg I | real number | mm |  |
| cox2 | Coxa II length | Coxa length of leg II | real number | mm |  |
| cox3 | Coxa III length | Coxa length of leg III | real number | mm |  |
| cox4 | Coxa IV length | Coxa length of leg IV | real number | mm |  |
| criw | Cribellum width | Width of cribellum or colulus | real number | mm |  |
| ctar | Claw tuft area | Projected area of adhesive foot pad (claw tuft) on leg IV | real number | mm^2^ |  |
| ctde | Claw tuft density | Density of adhesive foot pad (claw tuft) on leg IV, i.e. number of tenant setae per area unit | integer |  |  |
| eggs | Egg size | Diameter of an egg | real number | mm |  |
| eggv | Egg volume | Volume of an egg | real number | mm^3^ |  |
| epaw | Epigyne anterior plate width | Width of anterior border of epigyne plate | real number | mm |  |
| epcw | Epigyne central plate width | Width of central border of epigyne plate | real number | mm |  |
| eple | Epigyne length | Length of epigynal plate | real number | mm |  |
| eppw | Epigyne posterior plate width | Width of posterior border of epigyne plate | real number | mm |  |
| eyew | Eye region width | maximum width of eye region | real number | mm |  |
| fale | Fang length | Cheliceral fang length from base articulation to the tip (measured along the median arc) | real number | mm |  |
| fem1 | Femur I length | Femur length of leg I (measured between condyles) | real number | mm |  |
| fem2 | Femur II length | Femur length of leg II (measured between condyles) | real number | mm |  |
| fem3 | Femur III length | Femur length of leg III (measured between condyles) | real number | mm |  |
| fem4 | Femur IV length | Femur length of leg IV (measured between condyles) | real number | mm |  |
| l1le | Leg I length | Total length of one leg from the first (front) leg pair, excluding coxa and trochanter | real number | mm |  |
| l2le | Leg II length | Total length of one leg from the second leg pair, excluding coxa and trochanter | real number | mm |  |
| l3le | Leg III length | Total length of one leg from the third leg pair, excluding coxa and trochanter | real number | mm |  |
| l4le | Leg IV length | Total length of one leg from the fourth leg pair, excluding coxa and trochanter | real number | mm |  |
| met1 | Metatarsus I length | Metatarsus length of leg I (measured between condyles) | real number | mm |  |
| met2 | Metatarsus II length | Metatarsus length of leg II (measured between condyles) | real number | mm |  |
| met3 | Metatarsus III length | Metatarsus length of leg III (measured between condyles) | real number | mm |  |
| met4 | Metatarsus IV length | Metatarsus length of leg IV (measured between condyles) | real number | mm |  |
| ocdi | Ocular distance | Sum of diameters of one side of the caparace eyes (1 ALE, 1 PLE, 1 PME, 1 AME) | real number | mm |  |
| pat1 | Patella I length | Patella length of leg I (measured between condyles) | real number | mm |  |
| pat2 | Patella II length | Patella length of leg II (measured between condyles) | real number | mm |  |
| pat3 | Patella III length | Patella length of leg III (measured between condyles) | real number | mm |  |
| pat4 | Patella IV length | Patella length of leg IV (measured between condyles) | real number | mm |  |
| pled | PLE | Diameter of one posterior median eye | real number | mm |  |
| plsl | Spinneret PLS | Total length of posterior lateral spinneret (from base to tip) | real number | mm |  |
| pmed | PME | Diameter of one posterior lateral eye | real number | mm |  |
| pmsl | Spinneret PMS | Total length of posterior median spinneret (from base to tip) | real number | mm |  |
| ptal | Palpal tarsus length | Length of palpal tarsus in males | real number | mm |  |
| ptwi | Palpal tarsus width | Width of male palpal tarsus | real number | mm |  |
| scoc | Scopula cover | Relative area of the prolateral side of leg I segment(s) covered with hairy adhesive pad (scopula), excluding claw tufts | real number | mm^2^ |  |
| stle | Sternum length | Width of sternum at widest point | real number | mm |  |
| stwi | Sternum width | Length of sternum along the longitudinal axis | real number | mm |  |
| tale | Tegular apophysis length | Length of tegular apophysis on male bulbus | real number | mm |  |
| tar1 | Tarsus I length | Tarsus length of leg I (measured between condyles) | real number | mm |  |
| tar2 | Tarsus II length | Tarsus length of leg II (measured between condyles) | real number | mm |  |
| tar3 | Tarsus III length | Tarsus length of leg III (measured between condyles) | real number | mm |  |
| tar4 | Tarsus IV length | Tarsus length of leg IV (measured between condyles) | real number | mm |  |
| tawi | Tegular apophysis width | Width of tegular apophysis on male bulbus | real number | mm |  |
| tib1 | Tibia I length | Tibia length of leg I (measured between condyles) | real number | mm |  |
| tib2 | Tibia II length | Tibia length of leg II (measured between condyles) | real number | mm |  |
| tib3 | Tibia III length | Tibia length of leg III (measured between condyles) | real number | mm |  |
| tib4 | Tibia IV length | Tibia length of leg IV (measured between condyles) | real number | mm |  |
| tro1 | Trochanter I length | Trochanter length of leg I (measured between condyles) | real number | mm |  |
| tro2 | Trochanter II length | Trochanter length of leg II (measured between condyles) | real number | mm |  |
| tro3 | Trochanter III length | Trochanter length of leg III (measured between condyles) | real number | mm |  |
| tro4 | Trochanter IV length | Trochanter length of leg IV (measured between condyles) | real number | mm |  |
|  | Physiology |  |  |  |  |
| gluc | Glucose content | Amount of glucose in a wet mass | real number | µg/mg |  |
| ld50 | Venom toxicity | LD50 of venom on Drosophila prey | real number | nl venom/mg fly |  |
| pydr | Drought tolerance | Relative humidity the spider can tolerate | real number | % |  |
| pytl | Lower thermal limit | Temperature limit at which growth occurs | real number | °C |  |
| pymr | Resting metabolic rate | Oxygen consumption per time when inactive | real number | W |  |
| pysb | Submerging time | Time of surviving under water | real number | h |  |
| pytu | Upper thermal limit | Temperature limit at which growth occurs | real number | °C |  |
| prot | Protein content | Amount of proteins in a wet mass | real number | µg/mg |  |
| trig | Triglyceride content | Amount of triglycerides in a wet body mass | real number | µg/mg |  |
|  | Predation |  |  |  |  |
| cons | Consumption time | Time spent consuming certain prey (specified in Treatment variable) | real number | h |  |
| guil | Hunting guild | Ecological hunting guild according to Cardoso P, Pekár S, Jocqué R & Coddington JA 2011. Global patterns of guild composition and functional diversity of spiders. PloS One 6(6): e21710. | character |  | Sensing web weavers; sheet web weavers; space web weavers; orb web weavers; specialists; ambush hunters; ground hunters; other hunters. |
| klep | Kleptoparasitism | Occurrence of kleptoparasitism | character |  |  |
| nich | Trophic niche breadth | Levin's standardised index of niche breadth according to Hurlbert SH 1978. The measurement of niche overlap and some relatives. Ecology 59(1): 67-77. | real number |  |  |
| para | Paralysis latency | Time between attack and prey immobilisation | real number | min |  |
| prdi | Prey diversity | Shannon-Weaver index of diversity of captured prey as a measure of niche breadth | real number |  |  |
| prek | Overkilling | Proportion of prey items killed but not consumed | real number | % |  |
| prec | Prey capture | Mode of prey capture | character |  | Bite-and-release; grab-and-hold; wrapping; throwing silk; other |
| prem | Satiation | Number of prey items killed and consumed per certain time interval (specified in Treatment variable) | integer |  |  |
| preo | Prey order | Taxonomic order of an organism the spider preys on | character |  |  |
| prey | Prey stage | Developmental stage of prey organism | character |  | Egg; larva/caterpillar; pupa; imago |
| prsi | Prey size | Prey size (total body length) | real number | mm |  |
| stsp | Strike speed | Time to complete a predatory strike (start of strike to first bite) | real number |  |  |
| weba | Web area | Size of web projected in a 2-dimensional space | real number | cm^2^ |  |
| webb | Web building | Use of a web for prey capture (not a retreat) | character |  |  |
| webd | Web diameter | Linear dimension of a web | real number | cm |  |
| webt | Web type | Type of capture web | character |  | Orb web; cob web with gum-foot lines; sheet web; canopy web; space web; open tube; tube with trap door; tube with signalling lines; single line; other |
| webv | Web volume | 3-dimensional size of a web | real number | cm^3^ |  |
|  | Reproduction |  |  |  |  |
| coco | Coersive copulation | Presence of coercive mating indicated by causing injuries to the other sex | character |  |  |
| codi | Cocoon diameter | Maximum diameter of the cocoon | real number |  |  |
| coty | Courtship type | Sensual modality used during courtship (verbal description) | character |  |  |
| codu | Courtship duration | Time from starting the courtship to the beginning of copulation | real number | min |  |
| duma | Duration of mating | Total mating time | real number | min |  |
| eggm | Eggsac mass | Weight of an eggsac | real number | g |  |
| eggn | Number of eggs/sac | Number of eggs in a clutch (eggsac) / eggsac order | integer |  |  |
| egsn | Number of eggsacs | Total number of eggsacs produced by a female during her life | integer |  |  |
| eplu | Epigyne plugging | Mode of blocking access to the female epigyne | character |  | Excretion; embolus; none; other |
| fert | Fertility | Number of hatched offspring | integer |  |  |
| maca | Maternal care | Extent of maternal care | character |  | None; guarding egg sac; guarding egg sac and spiderlings |
| maph | Matriphagy | Presence of matriphagy (i.e., offspring consuming tissue of their mother) | character |  |  |
| mapo | Mating position | Type of a mating position according to Foelix R F. 2011. Biology of Spiders. 3rd ed. New York: Oxford University Press. | character |  | Type 1; type 2; type 3 |
| nuin | No. of insertions | Total number of insertions during copulation | integer |  |  |
| nupa | No. of partners | Total number of mated partners | integer |  |  |
| ovip | Oviposition | Time to oviposition (following the first mating) | real number | days |  |
| sexc | Sexual cannibalism | Presence of sexual cannibalism and the sex of cannibal | character |  | Female; male |
