# Supplementary material for: The World Spider Trait database: a centralized global open repository for curated data on spider traits
Source: Database (Oxford). 2021 Oct 20;2021:baab064. doi: 10.1093/database/baab064 (PMC8517500; doi:10.1093/database/baab064)
Supplement: baab064_Supp [file baab064_supp.zip › Table S2.docx]

**Table S2.** List of Methods. For each method there is an abbreviation, name and a short description.

| **Abbreviation** | **Method name** | **Description** |
| --- | --- | --- |
| bea | Beating | Capture by beating over net |
| cit | Citizen Science | Observation collected through citizen science |
| col | Colorimetry | Concentration assessment of a chemical compound in a homogenate |
| dis | Dissection | Obtained using dissection |
| exp | Expert-Base | Assessed based on expert opinion |
| fie | Field Observation | Observation performed in nature |
| fot | Photoeclector trapping | Capture by photoeclector |
| fun | Functional Response | Experiment of functional response |
| gut | Gut-Content Analysis | Molecular analysis of gut content |
| han | Hand Collection | Capture by individual hand sampling |
| kar | Karyology | Karyology on dissected tissue |
| lab | Laboratory Observation | Observation performed under laboratory conditions |
| mal | Malaise Trapping | Capture by Malaise traps |
| mic | Microscopic Measurement | Measurement done under microscope or in micro-photographs |
| mor | Morphometry | Length determination based on microscopy |
| mov | Movement Measurement | Measurements done using video-tracking software (e.g., Ethovision) |
| mul | Multiple data analysis | Analysis of results of former multiple studies |
| na | Not available | This information is not available |
| olf | Olactometry | Measurement done using olfactometer |
| pan | Yellow Pan Trapping | Capture by yellow pan traps |
| pho | Photographic Analysis | Analysis of photographs |
| pro | Protein content | Measurement of protein content using Bradford’s method |
| ptf | Pitfall Trapping | Capture by pitfall traps |
| res | Respirometry | Measurement done using respirometer |
| she | Shelter Trapping | Capture by shelters (e.g. bark bands) |
| sie | Sieving | Capture by sieving |
| sou | Sound Recording | Sound recorded by a recorder |
| spe | Spectrophotometric Measurement | Measurement done using spectrophotometer |
| suc | Suction trapping | Capture by a suction trap placed in the air |
| swe | Sweeping | Capture by sweeping net |
| tem | Transmission electron microscopy | Transmission electron microscopy using standard protocol for chemically fixed samples |
| the | Thermometry | Measurement done using temperature controlled chamber |
| tox | Toxicology | Toxicology bioassays |
| vac | G-VAC sampling | Capture by sucking up device. |
| ven | Venom potency test | Test of venom potency using a standardized protocol (specified in trait or notes) |
| web | Web Analysis | Analysis of the web content |
| wei | Weighing | Weighing on a lab scale (i.e. analytical balance) |
